# Implementation of a patient-reported outcome measure based digital journey and automation tool: A feasibility study in a surgical setting

**DOI:** 10.1097/EA9.0000000000000122

**Published:** 2026-06-22

**Authors:** Diana Castro Paupério, Filipe Freitas Pinto, Ana Dionísio, Henrique Coelho, Serafim Guimarães, Tomaz Crochemore, Donat R. Spahn, Joana Berger-Estilita, João Firmino-Machado

**Affiliations:** From the Departamento de Ciências Médicas, Universidade de Aveiro, Aveiro (DP, SG, JFM), Unidade Local de Saúde de Gaia e Espinho (DP, HC, SG, JFM), knok Healthcare, Ltd, Matosinhos, Portugal (AD), Faculdade de Medicina da Universidade do Porto, Porto, Portugal (FFP), Hospital Moriah, São Paulo, Brazil (TC), University of Zurich, Zurich (DRS), Institute for Medical Education, University of Bern, Mittelstrasse 43, Bern, 3012, Switzerland, Centre for Health Technology and Services Research, Faculty of Medicine, RISE-Health, University of Porto, Porto, Portugal, Department of Anesthesiology, University Medical Center Groningen, University of Groningen, Groningen, The Netherlands (JBE), Instituto de Saúde Pública da Universidade do Porto, Porto, Portugal (JFM)

## Abstract

**BACKGROUND:**

Patient blood management (PBM) has been shown to improve patient outcomes and optimise resource use. However, the systematic collection and analysis of patient-reported outcome measures (PROMs) remains challenging, limiting their integration into PBM pathways and into value-based healthcare (VBHC) strategies.

**OBJECTIVE(S):**

This study aims to evaluate the feasibility and adherence to an automated VBHC digital tool for PROMs collection in a population of cardiac surgical patients, within the context of PBM implementation.

**DESIGN:**

A single-centre, pragmatic, prospective feasibility study.

**SETTING:**

A tertiary Portuguese hospital considering data from 1 March to 6 September 2024. A total of 12 professionals were actively involved in the patient journey design.

**PATIENTS:**

A total of 221 patients (65.2% men, mean age 66.7 ± 9.9 years) with a scheduled cardiothoracic surgery were included.

Main outcome measures: Percentage of planned interactions successfully completed.

**RESULTS:**

A two-phase digital PROMs journey was implemented in less than 2 months. A total of 638 interactions were completed by 221 patients, resulting in a global completion proportion of 72.1%. 36-Item Short Form Survey Instrument (SF-36) and BPI questionnaires, completed by 77% of patients, results at 90 days compared to results at initial evaluation (SF-36 physical: t = -0.424, *P* = 0.681; mental: Z = 33.0, *P* = 1.000; Brief Pain Inventory (BPI) Pain Severity and Interference: Z = 23.0, *P* = 0.646). The PHQ-9, completed by 76% of patients, identified five severe and nine moderately severe depression cases. Educational content had a 73.9% reading completion proportion across 303 views.

**CONCLUSIONS:**

We showed the feasibility of implementing a digital tool to monitor patient PROMs, along with its adoption by patients and health professionals. This strategy has the potential to contribute to improving healthcare professionals’ motivation, access to real-world data, personalised monitoring, and allowing for faster implementation of corrective actions, with an impact on patients’ outcomes and costs.

**KEY POINTS**

- Anaesthesiology plays a critical role in delivering well tolerated and quality care, aiming to improve patient outcomes, reducing costs and incorporating a value-based healthcare (VBHC) perspective.
- A digitalised patient journey approach might overcome some of the traditional patient-reported outcome measurements (PROMs) implementation barriers and enable VBHC initiatives.
- Feasibility of automated PROMs collection practices digitalisation in cardiothoracic surgery was showed through the assessment of its real-life clinical setting implementation results.
- Very good operational results were found (with very high percentages of planned interactions successfully completed across various evaluations), which might lead to significant economic savings.
- PROMs are highly relevant to improve personalised care and VBHC through the identification of unattended or overlooked needs.

## Introduction

Anaemia affects over 2.9 billion individuals worldwide, with approximately 1.5 billion cases attributed to iron deficiency.^1^

Among patients scheduled for major surgery, the prevalence of pre-operative anaemia can reach up to 75%, depending on demographic and clinical factors.^2^ Pre-operative anaemia is an independent risk factor for peri-operative morbidity and mortality, and remains one of the primary drivers of red blood cell (RBC) transfusion.^3^ In addition, acute or chronic blood loss and bleeding disorders affect around 600 million people, further elevating the risk of peri-operative complications and the need for RBC transfusions. These factors, combined with intra-operative coagulopathy, emphasise the need to implement programs focused on improving the quality and safety of the quality of care provided, for example, peri-operative patient blood management (PBM).^4^

A large-scale pioneering study in Australia, including more than 600 000 patients, demonstrated significant benefits following the implementation of quality of care interventions, including a decrease in hospital mortality (-28%), in infection rates (-21%), myocardial infarction and stroke (-31%) and in transfusion rate (RBC: -41%; plasma: -47%; platelets: -27%).^5^ In addition, a 15% reduction in the hospital length of stay and estimated gross savings of 78 to 97 million US dollars were observed.^5^ Peri-surgical initiatives focused on similar factors have shown success in specific surgical areas, including cardiac surgery.^6^ In Portugal, a preliminary study by our group compared outcomes of two cohorts: one of patients undergoing cardiac surgery after PBM implementation and another from the same centre before PBM adoption.^4^ Results showed a statistically significant reduction in mean hospital length of stay (from 8.89 to 6.30 days) and a 63.2% decrease in the odds of RBC transfusion.^4^ This translated into an estimated cost saving of approximately 3000 euros per patient within the first year of PBM implementation.^4^

Despite these benefits, PBM implementation still faces significant barriers, including lack of stakeholder engagement, resistance to cultural change and operational complexity.^7^^–^^10^ The standardisation of clinical practices and the collection of patient-reported outcome measures (PROMs) pose further challenges to scaling value-based healthcare (VBHC) initiatives.^11^^,^^12^ Digital health technologies offer unprecedented opportunities to optimise healthcare workflows, reduce administrative burdens and improve operational efficiency. In the peri-surgical context, digitalisation may provide a streamlined approach to integrating complex care pathways, automating manual processes and enabling real-time data collection and monitoring. By replacing fragmented paper-based systems with automated digital workflows, clinicians can focus more on delivering personalised care rather than managing logistical challenges.^13^ In addition, real-time data visualisation through dynamic dashboards allows healthcare providers to make informed, timely decisions based on accurate patient data. Digital platforms not only enhance clinical team motivation by reducing repetitive tasks but also facilitate better resource allocation, ultimately improving patient outcomes and advancing peri-operative care.

Health informatics and robust information systems are, therefore, critical enablers for successful high-quality and well tolerated care, and VBHC adoption.^14^

The current study evaluates the feasibility of digitalising PROMs collection practices within a hospital setting, using cardiothoracic surgery as the setting assessing the preliminary operational outcomes of the digital platform implementation and focusing on its potential to streamline workflows, improve data collection, and enhance clinical outcomes. The digital journey success was assessed using a combination of primary and secondary outcome measures. The primary outcome was defined as the percentage of planned interactions completed, as a key indicator of patient adherence and engagement with the digital journey platform.

## Materials and methods

### Ethics

Ethical principles from the Declaration of Helsinki were followed and informed consent for data use was obtained from all patients. Ethical approval (196/2023-1) was provided by the Ethics Committee of Unidade Local de Saúde Gaia e Espinho (Vila Nova de Gaia, Portugal), on 24 October 2023 (Head of Ethics Committee: Dr. Paula Fernandes).

Data security and confidentiality were carefully ensured. Patient data were collected through the hospital's standard electronic medical record platforms, which comply with the appropriate storage and data transmission requirements. PROM collection and patient notifications were conducted using the commercial digital platform *knok* (knokcare, Portugal, www.knokcare.com). The platform holds ISO 27001 certification and complies with all the General Data Protection Regulation requirements and cybersecurity standards.

### Study design

This is a single-centre, pragmatic, prospective feasibility study conducted at Unidade Local de Saúde Gaia e Espinho, a tertiary hospital in Portugal. Data were collected over six months, from 1 March 2024 to 6 September 2024.

### Patients

Patients were identified through the hospital database. Inclusion criteria were age more than 18 years to focus on adult patients; registration for cardiothoracic surgery at the tertiary hospital, regardless of the specific procedure type; and completion of a pre-anaesthesia visit for standardised data collection. Surgical procedures include – but are not limited to – valve replacement or repair, coronary artery bypass grafting (CABG), combined procedures, aortic surgery, and urgent or emergent cases.

Exclusion criteria included incomplete or missing pre-anaesthesia records; emergency surgeries due to insufficient pre-operative data; and cognitive impairments or communication barriers affecting informed consent or data reliability.

In practice, two groups of patients were considered. The first group featured patients referred by the surgical team for pre-anaesthetic evaluation are automatically enrolled in the digital PROMs collection journey during their pre-anaesthesia consultation. These patients follow the ‘pre-habilitation path’, which enables early optimisation prior to surgery. The second group comprised patients who are not referred for pre-anaesthetic assessment – due to time constraints or logistical reasons – or urgent/emergent cases. These patients are enrolled on the day of surgery, initiating the ‘surgical path’. In both cases, data collection begins prospectively upon admission.

Since the implementation of the digital PROMs collection journey, all adult patients undergoing cardiothoracic surgery – elective, urgent or emergent – have been systematically identified.

### Data collection

The automated digital PROMs collection pathway featured two groups of outcome measures. This study focuses on dimensions such as anxiety, depression, fatigue and quality of life. Collection of these data is proposed to patients during pre-anaesthesia consultation or postoperative follow-up. Participation in PROMs collection was voluntary and could be declined.

In parallel, clinician-reported outcome measures (CROMs) – including haemoglobin (Hb), iron studies, coagulation profiles, transfusion data and complications – were collected automatically through integration with the hospital information system. This occurred under the scope of institutional consent provided upon hospital admission, as part of standard clinical care.

### Outcome measures

The digital journey success was assessed using a combination of primary and secondary outcomes:

#### Primary outcome

(1) Percentage of planned interactions successfully completed.

#### Secondary outcomes

(1) Number of professionals involved, their specialisations and overall motivation.
(2) Time taken to fully digitalise the patient journey.
(3) Platform adherence
  (a) Number of patients enrolled per month.
  (b) Number of completed interactions.
  (c) Time from delivery until completion of interactions.
  (d) Proportion of educational content accessed by patients.
  (e) Average time spent engaging with educational content.
(4) Evolution of PROMs results across timepoints.

### Patient-reported outcome measures collection

A set of PROMs was collected through the digital platform. PROMs were automatically sent to patients via customisable email and SMS notifications containing hyperlinks to the platform. Patients completed the questionnaires remotely, except during the initial evaluation, where they could complete them at the hospital.

The following PROMs are included and the rationale for their adoption is described:

- 36-Item Short Form Survey Instrument (SF-36): The SF-36 assesses health-related quality of life (HR-QoL) and is composed of 36 items evaluating eight domains of general health status, grouped into two main components: physical and mental. Lower scores represent a poorer perception of HR-QoL by patients. The response time ranges between 5 and 10 min^15^ (median for adults aged 65 and above: 8 min^16^). It is quite relevant in surgical settings as factors such as blood loss and anaemia can negatively impact energy, function and mental health. SF-36 helps detect these impacts by assessing HR-QoL.
- Brief Pain Inventory Short Form (BPI-SF): The BPI-SF evaluates pain severity and impact on daily activities. It includes nine items assessing current and recent pain levels (Pain Severity Index) and the interference with daily functions and activities (Pain Interference Index).^17^ The average response time is 5 min.^17^ BPI-SF is a very relevant PROM as pain can be a direct consequence of surgery and can be exacerbated by anaemia or blood loss. Effective peri-surgical care may reduce the need for transfusion-related interventions, improving rehabilitation and overall patient satisfaction.
- Patient Health Questionnaire (PHQ-9): The PHQ-9 comprises nine items that address the DSM criteria for depressive disorders and can be used to measure depression severity.^18^ Kroenke *et al*.'s^19^ cut-offs were used to classify depression severity into five levels: minimal (scores 0 to 4), mild (5 to 9), moderate (10 to 14), moderately severe (15 to 19) and severe (20 to 27). The average response time is 5 min for adults aged 65 years and older.^20^ As anaemia and major surgery are known contributors to postoperative depression, psychological recovery may be affected. Monitoring depression can help evaluate if adequate care delivered helps maintain mental health by minimising physiological stress and improving recovery trajectories.
- Use of health services: Patients reported whether they sought healthcare services outside the hospital after surgery. If yes, they were asked about the frequency and type of service (e.g. public/private emergency care, public/private medical appointments). As one aim of high-quality and well tolerated care is to reduce postoperative complications and readmissions by improving patient outcomes, a reduction in unplanned healthcare use suggests its effectiveness.
- Infection screening report: Patients reported whether they had taken antibiotics, anti-inflammatory or antipyretic drugs to screen for potential infections after surgery. Due to blood transfusions increasing infection risk, peri-surgical and surgical practices that reduce or eliminate transfusions may lower postoperative infection rates. Self-reported use of antibiotics or antipyretics may indicate subclinical or undiagnosed infections, giving insight into the indirect outcomes of blood management strategies.
- Return to work: Patients indicated whether they had returned to work at predefined postoperative timepoints. Considering high-quality and well tolerated care delivery aims to accelerate functional recovery and reintegration into normal life, faster return to work indicates better functional recovery, possibly due to better managed anaemia and reduced complications. This real-world functional outcome reflects overall postoperative success and quality of recovery, directly tied to the effectiveness of clinical interventions.

**Fig. 1:**
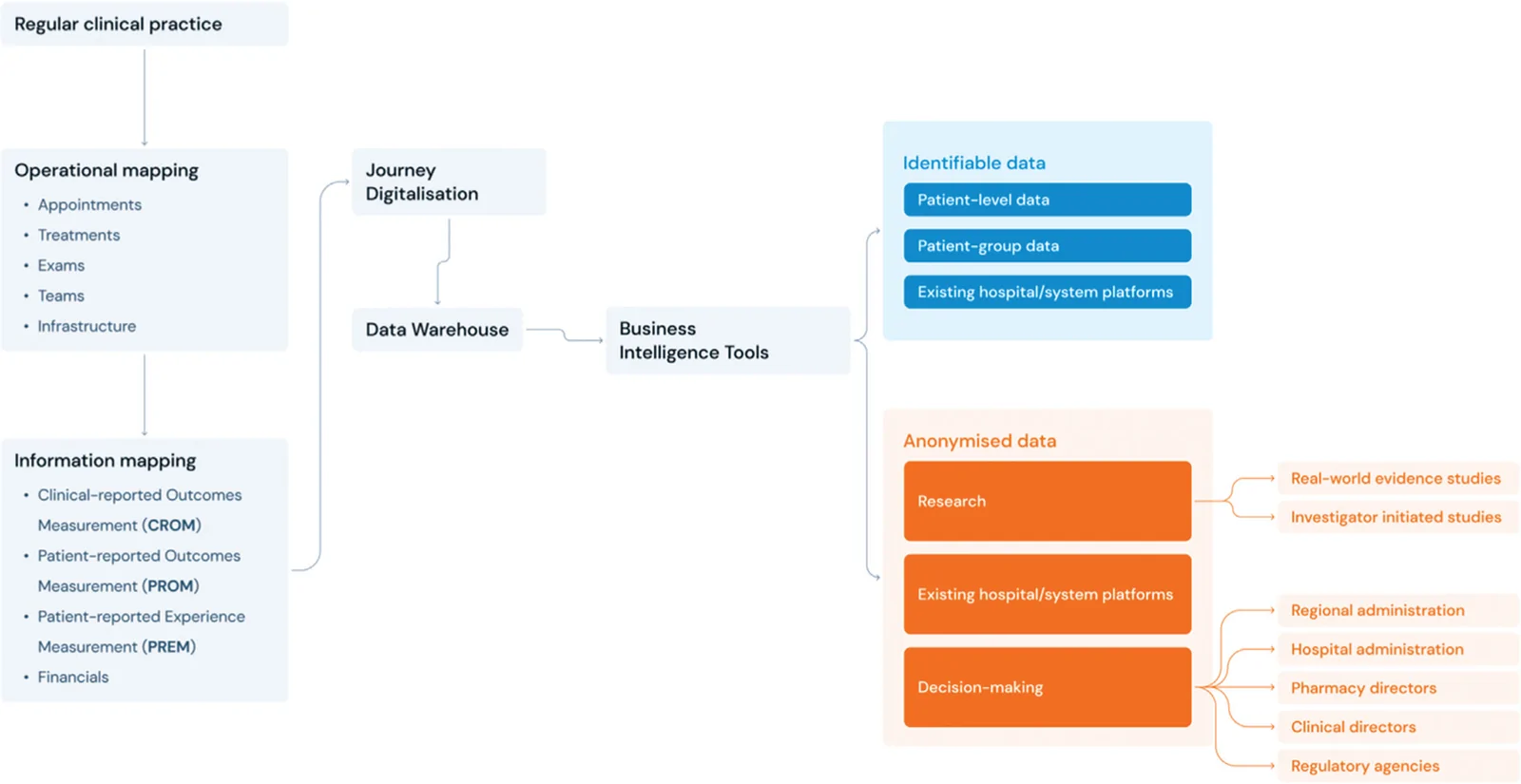
Patient journey mapping protocol.

By default, interactions were prompted once per appointment, as described in Fig. 1. These moments are described in Table 1 as ‘planned’ interactions. In addition, some on-demand interactions were prompted by the physician. Questions were presented in a standardised order, and similarly to all patients. Only totally completed interactions/surveys were considered as finished. Interactions partially completed were considered as ‘pending’.

**Table 1:** Planned and unplanned completed interactions in each phase of the journey

	Number of patients enrolled	Number of completed interactions	% of completed interactions
Informational (planned)			
Welcome & educational content	221	221	100
Pre-habilitation Path (planned)			
Initial evaluation	214	157	73.4
Surgery path (planned)			
Pre-operative	14	9	64.3
Follow-up at 40 days	41	30	73.2
Follow-up at 90 days	22	13	59.1
On demand (unplanned)			
Questionnaires		18	
Notifications		190	

### Patient journey

Educational Material: At the beginning of the journey, a six-page booklet was provided to patients. This resource was designed to explain peri-surgical and patient blood management (PBM) concepts using layperson-friendly language, ensuring accessibility across different literacy levels. The booklet covered PBM definition, objectives and benefits, normal/abnormal Hb concentrations, an explanation of pre-surgical procedures and medications, strategies to minimise blood loss during surgery and the patient's role in the process (Supplementary Digital File 1). The aim of this support document was to help patients quickly revise or understand the main concepts and terms regarding their clinical journey. It served to improve basic literacy on the care provided and provide an additional source of contacts to seek further assistance in case of doubts or complications. To improve accessibility and readability, this document featured large typeface and simple graphical design elements.

Patient Journey Mapping: Twelve professionals with diverse expertise – including anaesthesiology, haematology, public health, healthcare management and digital health – collaboratively developed the patient journey and PROMs mapping protocol (Fig. 1), designed to align with standard care practices.^21^ The operational and information mapping were carried out by clinical teams, including anaesthesiologists, haematologists, nursing staff and epidemiologists. The technology partner supported this process by digitalising the patient journey, to ensure seamless data collection, compliance with data protection standards and real-time data analysis.

**Fig. 2:**
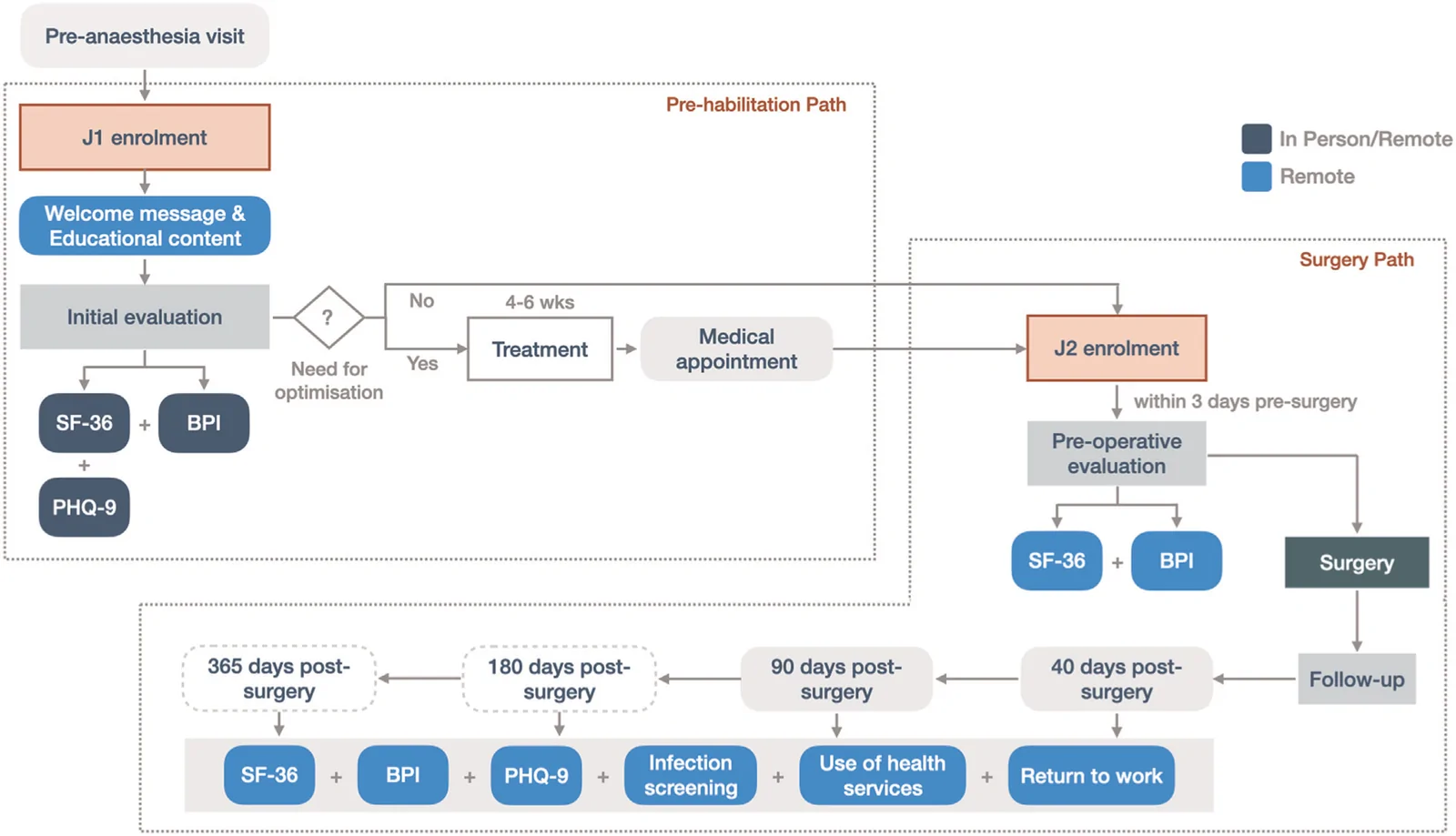
PBM patient journey flowchart.

Patient Journey Description: Figure 2 shows the patient journey diagram. The journey was divided into two paths. This division was based on the technical configuration and temporal anchoring of the digital platform to ensure appropriate data capture and sequencing, and not on clinical considerations.

The pre-habilitation path refers to patients who enter the journey prior to surgery, typically through a structured pre-anaesthetic consultation. The journey begins with a medical appointment and an evaluation using the SF-36, BPI and PHQ-9 questionnaires. In this case, the platform initiates pre-operative data collection (e.g. haematinic indices, anaemia correction interventions, patient-reported symptoms) and schedules time-sensitive follow-up tasks. The anaesthesiologist/haematologist assesses the need for blood optimisation based on blood test results, including Hb, transferrin saturation, ferritin, C-reactive protein, vitamin B12 and folic acid concentrations. When the Hb concentration is inferior to the cut-off (Hb <13 g dl^−1^),^2^ patients are considered eligible for PBM. When Hb concentration is within the normal range but below 14.5 g dl^−1^ (according to the European Society of Cardiology guidelines),^22^ the decision for PBM eligibility considered the remaining blood parameters. Patients needing blood optimisation were treated for 4 to 6 weeks, followed by a medical appointment. If optimisation was unnecessary, the patient moved directly to the surgery path.

The surgery path, by contrast, is designed for patients who enter the digital journey after surgery has occurred, either due to urgency, absence of prior referral or logistical constraints. Acknowledging that the pre-operative window has closed, the platform immediately initiates postoperative tracking and follow-up, including PROMs and clinical outcomes. This path started with one pre-operative evaluation conducted within 3 days before surgery, consisting of the SF-36+BPI questionnaires.

Post-surgery, follow-up PROMs included SF-36, BPI, PHQ-9, infection screening report, use of health services and return to work and were administered remotely at 40 and 90-day post-surgery. Two future follow-up timepoints are planned (180 and 365-day post-surgery).

This structure of the follow-up considering these two paths, from a technical standpoint, provided three key benefits. First, it allowed the platform to anchor follow-up questionnaires (PROMs and CROMs) to the correct postoperative time points (e.g., days 40, 90, 180 and 365), even if no pre-operative phase was digitally documented. Second, it ensured the appropriate triggering of postoperative modules, such as CTCAE and additional quality-of-life instruments, which differ from those used pre-operatively. Lastly, it better reflected the evolution of the patient's journey, informing the digital system that surgery has occurred so it can shift from a preparatory optimisation logic to a recovery and outcome-monitoring mode. Overall, both paths ultimately converge in a continuous peri-surgical journey.

Clinical consultations continued to follow the standard of care. CROMs, namely blood test results, were collected as part of routine care throughout the patient journey and automatically compiled and presented in the digital platform.

### Data collection, presentation and statistics

Clinical data, including CROMs and demographic information, were imported directly from the hospital's information system. PROMs were collected using knok's digital platform. All data were integrated into Google's serverless data warehouse, BigQuery (Mountain View, California, USA), where they were cleaned and harmonised prior to analysis.

For clinical teams, the results were visually presented in dynamic dashboards, in an intuitive format that facilitated real-time monitoring and informed decision-making throughout the care process.

In this study's context, operational metrics and PROMs responses were analysed. Categorical variables were summarised as frequencies and percentages. Continuous variables were reported as means and standard deviations for normally distributed data, or as medians and interquartile ranges [IQR] for skewed distributions. The normality of data distribution was assessed using the Shapiro–Wilk test or by examining skewness and kurtosis values.

To compare PROMs results between two different timepoints (e.g. initial evaluation vs. pre-operative assessment, 40-day follow-up and 90-day follow-up), a paired samples *t*-test or its non-parametric equivalent Wilcoxon signed-rank test was used accordingly. *P* was considered significant when less than 0.05. Statistical analyses were conducted using the Python programming language (https://www.python.org/).

## Results

Data from 221 patients (65.2% men) undergoing cardiothoracic surgery were collected. Patients had a mean age of 66.7 ± 9.9 years, the majority (76.5%) being 60 years old or above. To date, the refusal rate for PROMs is under 5%, with most refusals occurring in elderly or frail patients or in situations of cognitive or emotional overload.

### Operational success

The digital patient journey implementation was achieved in less than 2 months, including five key phases: team brainstorming; literature review on relevant PROMs; definition of the journey pathway (including questionnaires, SMS and e-mail notifications and timepoints); integration of information systems; and digitalisation of the clinical workflow. The digitalisation process – that is, integration of the clinical flowchart into the platform – was completed in less than 2 h. Appointments in which the digital pathway was used took no longer than the previously used methods.

**Fig. 3:**
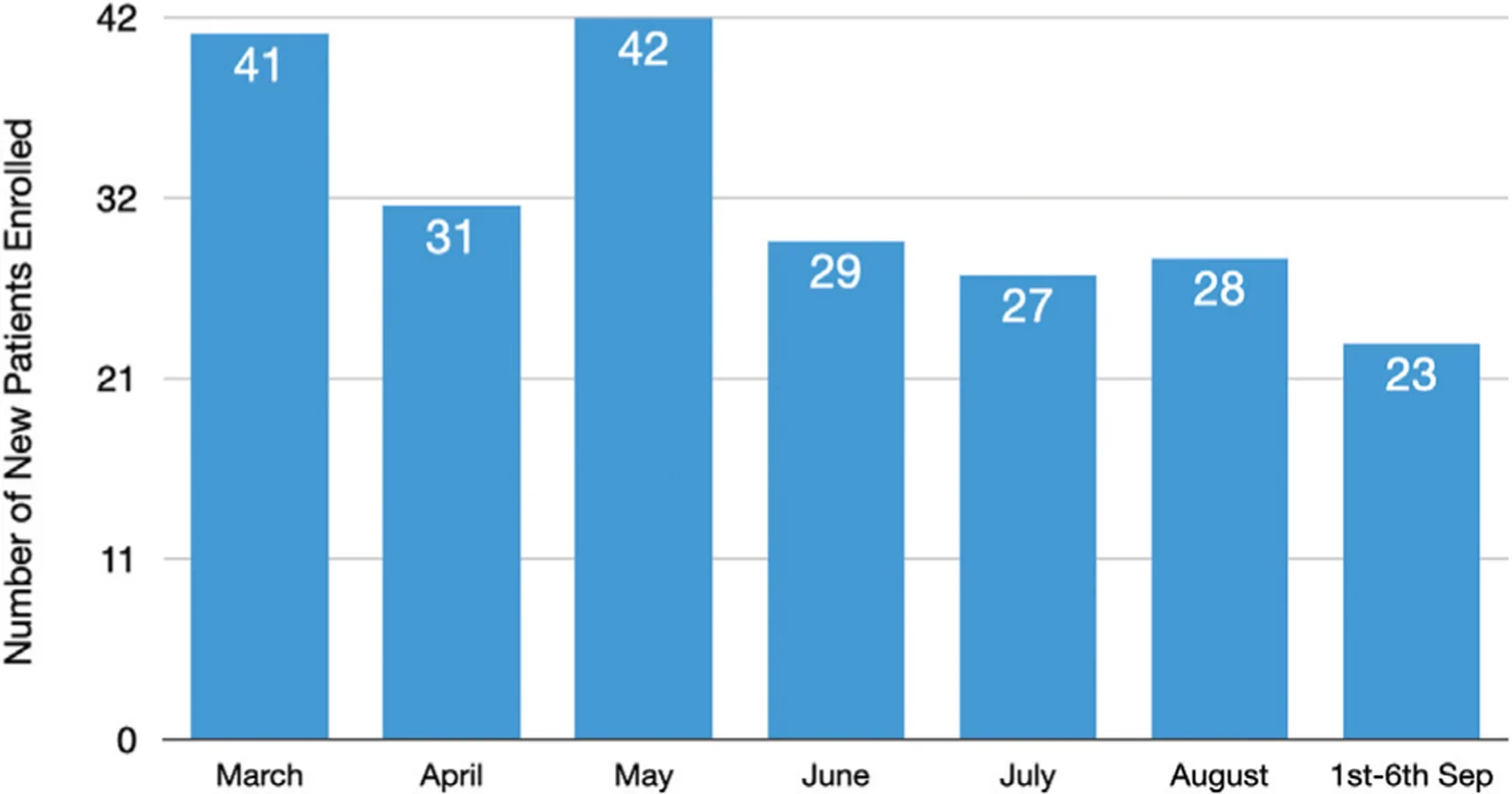
Monthly distribution of new patient enrolments throughout the study period.

Two hundred twenty-one new patient enrolments were recorded (the first enrolment per participant). The number of new patients enrolled per month ranged from 27 (July) to 42 (May) (Fig. 3). In September, in only 6 days, 23 patients were enrolled for the first time.

Forty-two patients were enrolled twice, as they were included in both paths of the described clinical journey. Considering both first-time and second-time enrolments, a total of 263 enrolments was observed (‘Pre-habilitation Path’: *n* = 218; ‘Surgery Path’: *n* = 45).

Patients were enrolled in different phases of the journey to match their stage in the clinical care process. Nevertheless, most of the patients were enrolled during their initial evaluation, ensuring early integration into the digital journey.

Throughout the study period, 638 interactions were completed. These included welcome messages, educational content delivery, planned and on-demand PROM assessments and system notifications. The distribution of these interactions across the different phases of the patient journey is summarised in Table 1.

The overall completion proportion of planned PROMs interactions was 72.1%, with a slightly higher completion proportion observed in the ‘Pre-habilitation Path’ (73.4%) compared to the ‘Surgery Path’ (68.1%). The maximum completion proportion was observed on the initial evaluation (*n* = 157; 73.4%) and the minimum on the 90-day follow-up (*n* = 13; 59.1%).

The digital tool allowed clinicians to filter PROM completion proportions by individual patients, offering insights into each patient's level of engagement. This feature enabled clinicians to identify patients with low engagement levels and proactively address them through tailored follow-up interactions, ensuring better adherence to the PROM schedule.

Mean time from delivery until completion of PROMs interactions ranged from 12 h (pre-operative assessment) to 193 h at (40-day follow-up).

In addition to PROMs, the platform allowed for real-time inspection of blood parameters (CROMs). Clinicians could quickly assess key indicators (including Hb concentration, transferrin saturation, ferritin, C-reactive protein, vitamin B12 and folic acid levels) that were presented in an intuitive colour-coded format, enabling rapid interpretation. They informed decision-making regarding individual PBM eligibility at any point in the care pathway.

### Patient-reported outcomes

**Fig. 4:**
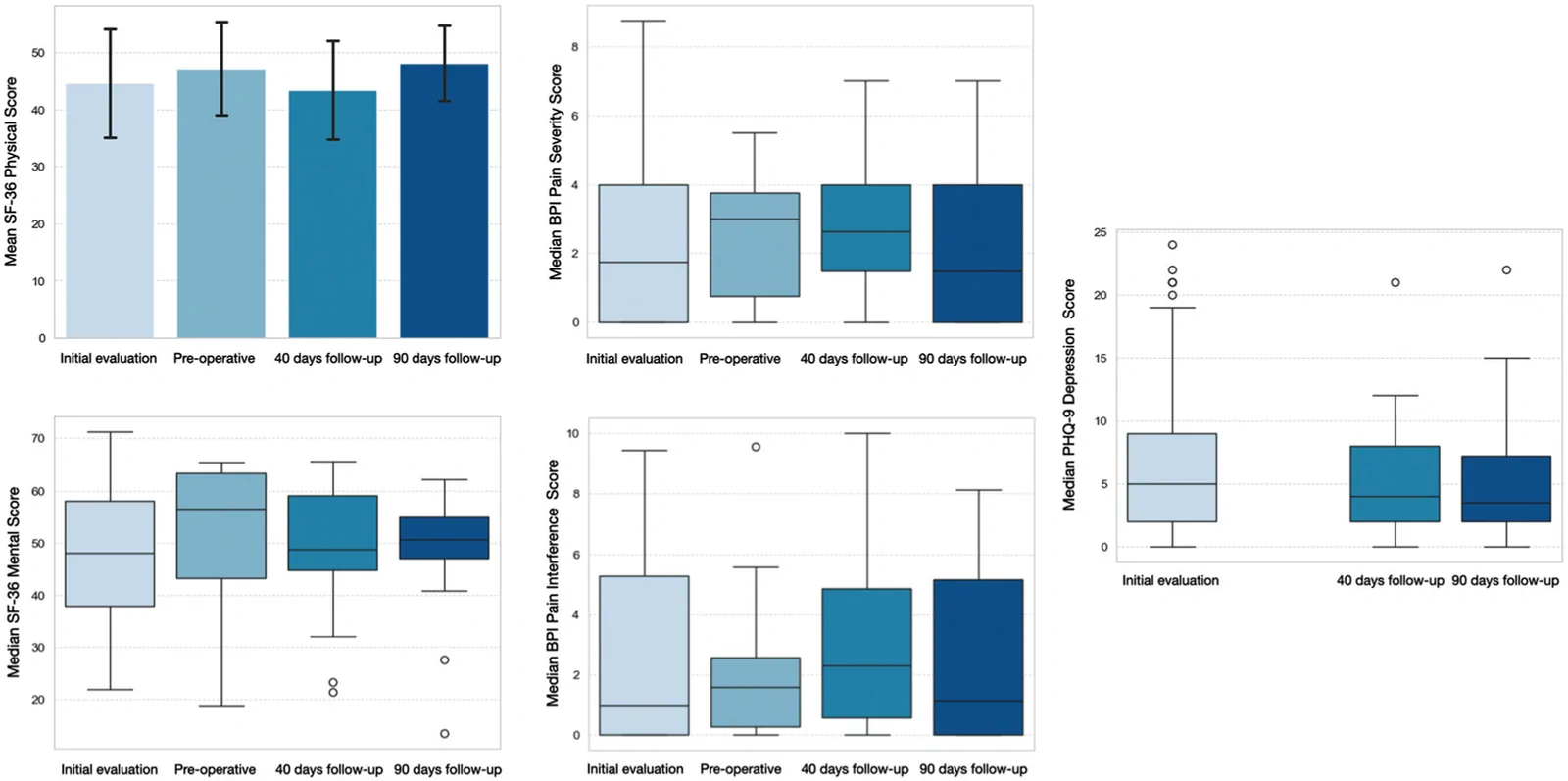
Evolution of the 36-Item Short Form Survey Instrument (SF-36), Brief Pain Inventory Short Form (BPI) and Patient Health Questionnaire (PHQ-9) scores across timepoints.

The evolution of PROMs was assessed. PROMs’ results in each journey phase are summarised in Table 2 and Fig. 4.

**Table 2:** Mean/median results of patient-reported outcomes in each phase of the journey

	36-Item Short Form Survey Instrument (SF-36)			Brief Pain Inventory Short Form (BPI-SF)			Patient Health Questionnaire (PHQ-9)	
	*n* (%)^a^	Physical (mean ± SD)	Mental (median [IQR])	*n* (%)^a^	Pain severity (median [IQR])	Pain interference (median [IQR])	*n* (%)^a^	Depression severity (median [IQR])
PLANNED INTERACTIONS								
Pre-habilitation path								
Initial evaluation	157 (71.0)	44.6 ± 9.5	48.0 [20.1]	157 (71.0)	1.75 [4.0]	1.00 [5.3]	153 (69.2)	5.00 [7.0]
Surgery path								
Pre-operative	9 (4.1)	47.2 ± 8.2 *t* = 0.051; *P* = 0.961^b^	56.5 [20.0] *Z* = 5.0; *P* = 0.078^b^	9 (4.1)	3.00 [3.0] *Z* = 4.0; *P* = 0.172^b^	1.57 [2.3] *Z* = 10.0; *P* = 0.499^b^		
Follow-up 40 days	30 (13.6)	43.4 ± 8.7 *t* = 0.898; *P* = 0.378^b^	48.8 [14.2] *Z* = 133.0; *P* = 0.643^b^	30 (13.6)	2.63 [2.5] *Z* = 84.5; *P* = 0.103^b^	2.29 [4.3] *Z* = 114.5; *P* = 0.697^b^	29 (13.1)	4.00 [6.0] *Z* = 94.5; *P* = 0.694^b^
Follow-up 90 days	13 (5.9)	48.1 ± 6.6 *t* = -0.424; *P* = 0.681^b^	50.7 [8.0] *Z* = 33.0; *P* = 1.000^b^	13 (5.9)	1.50 [4.0] *Z* = 23.0; *P* = 0.646^b^	1.14 [5.1] *Z* = 23.0; *P* = 0.646^b^	12 (5.4)	3.50 [5.3] *Z* = 15.5; *P* = 0.723^b^
UNPLANNED INTERACTIONS								
On demand	17 (7.7)	41.5 ± 7.6	44.9 [20.1]	17 (7.7)	3.25 [4.0]	2.86 [7.1]	9 (4.1)	2.00 [2.0]
	**Use of health services**		**Infection report**		**Return to work**			
	*n* (%)	Yes:No	*n* (%)	Antibiotics:None	*n* (%)	Yes:No		
Surgery path								
Follow-up at 40 days	30 (13.6)	9:21	9 (4.1)	4:5	30 (13.6)	6:24		
Follow-up at 90 days	13 (5.9)	3:10	3 (1.4)	2:1	13 (5.9)	4:9		

a The number *n* of unique patients who filled the questionnaire at each timepoint, and their proportion (%) in relation to the total sample of 221 patients included in the whole journey.

b *P*-values represent comparisons between two timepoints (initial evaluation versus pre-operative, follow-up at 40 days and follow-up at 90 days, respectively).

Scores are represented by mean ± SD or median [IQR], as appropriate.

#### SF-36 physical and mental components

The SF-36 questionnaire was completed 209 times during planned interactions.

The overall mean score for the physical component was 44.8 ± 9.2, and the median score for the mental component was 48.8 [IQR 19.1]. At the initial evaluation, the mean physical score was 44.6 ± 9.5, increasing slightly to 48.1 ± 6.6 at 90-day follow-up (mean difference: -1.414; 95% CI -8.85 to 6.02; *P* = 0.681). The lowest physical score was observed at 40-day follow-up (43.4 ± 8.7), and the highest at 90-day follow-up (48.1 ± 6.6).

For the mental component, the median score at the initial evaluation was 48.0 [IQR 20.1], compared to 50.7 [IQR 8.0] at 90-day follow-up (*Z* = 33.0; *P* = 1.000). The lowest mental score occurred at the initial evaluation (48.0 [IQR 20.1]) and the highest at the pre-operative assessment (56.5 [IQR 20.0]). Unplanned SF-36 assessments triggered by clinical concerns showed lower scores in both physical and mental components compared to planned assessments.

#### Brief pain inventory (BPI-SF)

The BPI-SF questionnaire was completed 209 times during planned interactions. The overall median score for Pain Severity Index was 2.00[IQR 4.0], and for Pain Interference Index it was 1.29[IQR 5.1]. At the initial evaluation, the Pain Severity Index had a median score of 1.75[IQR 4.0], decreasing slightly to 1.50[IQR 4.0] at 90-day follow-up (Z = 23.0; *P* = 0.646).

The Pain Interference Index median score was 1.00[IQR 5.3] at initial evaluation, increasing to 1.14[IQR 5.1] at 90-day follow-up (Z = 23.0; *P* = 0.646). The lowest Pain Severity Index was observed at 90-day follow-up (1.50 [IQR 4.0]), while the highest was at pre-operative evaluation (3.00 [IQR 3.0]). The Pain Interference Index was lowest at initial evaluation (1.00 [IQR 5.3]) and peaked at 40-day follow-up (2.29 [IQR 4.3]). Unplanned BPI-SF assessments showed higher scores for both indices compared to planned assessments.

#### Patient health questionnaire-9 (PHQ-9)

The PHQ-9 questionnaire was completed 194 times during planned interactions, with an overall median score of 5.00[IQR 7.0]. At the initial evaluation, the median score was 5.00[IQR 7.0], decreasing to 3.50[IQR 5.3] at 90-day follow-up (*Z* = 15.5; *P* = 0.723). The highest PHQ-9 median score was observed at initial evaluation (5.00[IQR 7.0]), while the lowest was at 90-day follow-up (3.50[IQR 5.3]).

An automatic alert system flagged patients at risk based on their PHQ-9 scores. At the initial evaluation, five patients scored for severe and nine patients for moderately severe depression.

#### Use of health services

The Use of Health Services questionnaire was completed 43 times. At 40-day follow-up (*n* = 30), 21 patients (70%) did not require external health services. Among the remaining patients, four (13.3%) required services once, one (3.3%) three times and four (13.3%) more than three times. At 90-day follow-up (*n* = 13), two patients (15.3%) used external services once, and one (7.7%) more than three times. All external visits were to public health services, predominantly urgent care units.

#### Infection screening

The infection screening report was completed 12 times. At 40-day follow-up, four out of nine patients reported antibiotic use. At 90-day follow-up, two out of three patients reported antibiotic use. No patients reported using anti-inflammatory or antipyretic medications.

#### Return-to-work

The Return-to-Work questionnaire was completed 43 times. At 40 days after surgery, 20% (*n* = 6) of patients had returned to work. By 90-day follow-up, this percentage increased to 30.8% (*n* = 4), representing a proportional increase of 53.8% (95% CI -90.6 to 198.3).

#### Individual PROM evolution

Patient-level PROMs data were visualised in individualised graphs showing the evolution of scores over time (Supplementary Digital File 2). Clinicians used these visualisations during consultations to inform and tailor care plans based on individual patient trajectories.

### Educational content adherence

The content was opened 303 times, with an average visit duration of 102 s (approximately 1 min and 42 s), and 75% of the openings rated above 131.5 s (approximately 2 min and 11 s). The average completion proportion (reading through all the pages) was 73.9%, with 50% of the patients reading through the document. Each patient opened the educational content for an average of 1.15 times.

The patients’ experience across the different stages of interaction (from the onboarding to the platform to PROMs collection) is presented in Supplementary Digital File 3.

## Discussion

This feasibility study demonstrates the successful digitalisation of the PROMs collection program for cardiothoracic surgery, highlighting its practicality, robust engagement and high adherence rates among clinical teams and patients.

The digital pathway facilitated the collection of PROMs, uncovering unmet needs such as managing pre-operative depression, a prevalent issue in cardiac surgery patients. This aligns with findings from Kaptain *et al*.,^23^ who emphasised the importance of personalised, patient-centred approaches in surgical pathways, towards a VBHC approach.

### The role of anaesthesia in automated digital patient-reported outcome measures implementation

Anaesthesiologists are uniquely positioned to impact VBHC in surgical patients, through their roles in pre-operative optimisation, intra-operative management and postoperative recovery.^24^^,^^25^ The rapid deployment of the automated digital PROMs collection program within 2 months and the active involvement of anaesthesiologists underscores their central role and motivation in aligning clinical workflows with VBHC models.

This study also reaffirms their contributions, particularly in interpreting CROMs, such as Hb and micronutrients levels and inflammatory markers, in collaboration with Haematology. This multidisciplinary approach echoes the findings of Grocott *et al*.,^26^^,^^27^ who advocated for risk-adapted, patient-centred peri-operative pathways.

### Digitalisation streamlined workflow and reduced administrative burden

Implementing the digital PROMs platform simplified complex processes, including data collection, patient tracking, and real-time analysis. By automating manual tasks, the platform reduced the administrative workload for clinicians and improved team motivation. Real-time, structured data visualisation enabled clinicians to quickly identify PBM-eligible patients and focus on delivering personalised care rather than managing logistical challenges.

Prior to the implementation of the digital platform, at the Unidade Local de Saúde Gaia e Espinho, PROMs – such as anxiety, depression and health-related quality of life – were previously collected in only a small number of patients and exclusively at the beginning of the follow-up period, typically during the pre-anaesthesia consultation. Notably, it took the same hospital 4 years and over 1000 h to manually collect PROMs for just 52 patients, highlighting the unsustainable burden of non-digital workflows. This limitation was explicitly addressed in prior work, ‘Prevalence and risk analysis for depression after open-heart valve replacement surgery’, where PROM data were collected only at baseline and could not be followed longitudinally.^28^

Similarly, prior to the implementation of the digital platform, even CROMs – such as Hb levels, transferrin saturation, ferritin, vitamin B12, folate and inflammatory markers – were manually extracted from disparate sources within the hospital's electronic medical records. This manual data curation was both labour-intensive and time-consuming. For example, it took our team over 5 years to manually extract and process clinical data from approximately 2000 patients as part of a foundational cohort.

The transition to a digital collection journey has since addressed these limitations by enabling automated and structured registry of both PROMs and CROMs, allowing for real-time monitoring, improved clinical decision-making and enhanced integration with value-based care frameworks.

### Patient engagement and adherence patterns

Engagement with the digital journey remained high, with overall PROM completion proportion reaching 72.1%. Adherence was notably higher in the ‘Pre-habilitation Path’ compared to the ‘Surgery Path’, suggesting that earlier integration into the care pathway fosters better engagement. Completion proportion declined 90-day post-surgery, possibly due to patient fatigue or reduced motivation.

Despite concerns about a ‘digital divide’ in older demographics,^29^ 76.5% of participants were aged 60 years and above and actively engaged with the platform, mirroring findings from Grocott *et al*.^27^ and Kaptain *et al*.,^23^ who noted growing acceptance of digital tools among older patients.

Importantly, this study included elderly cardiac surgery population – traditionally considered digitally excluded – using simple tools such as SMS or email, and to assess their adherence to digital PROMs and educational interactions. Our results support that this group can indeed interact meaningfully with digital health tools across the surgical pathway.

All the patients who started the follow-up in this study remained registered in the Unidade Local de Saúde Gaia Espinho. Therefore, patient transfer did not influence their adherence and engagement in this study.

### Improved patient outcomes through digital tools

The integration of digital tools enabled streamlined early anaemia detection through CROMs automatic presentation and workload automation. This is particularly important as this condition affects approximately 20% of the Portuguese population.^4^

Digital PROMs enabled timely psychological interventions by identifying patients at risk of depression, supporting literature that emphasises the critical role of mental health monitoring in peri-operative success.^23^^,^^30^^,^^31^ A nonsignificant trend for improvements in SF-36 scores and declining pain severity over time was observed. Similar patient engagement and outcome improvement trends have been noted in studies on enhanced recovery after surgery (ERAS) and peri-operative pathways.^23^^,^^25^

These results are consistent with Müller *et al*.'s findings,^25^ which demonstrated the value of clinical pathways in reducing resource utilisation and improving patient outcomes.

In addition, while the focus of this study was the feasibility and adherence to a digital, we recognise the importance of a detailed characterisation of patient and surgical variables, as well as PBM-related clinical outcomes. Variables such as anaemia diagnosis, iron and vitamin treatment, erythropoiesis-stimulating agents and transfusion practices are crucial to evaluate PBM effectiveness comprehensively.

Although the current feasibility study was not powered to assess clinical outcomes, we are pleased to report that many of these parameters are already being collected automatically and systematically via the digital platform and are being explored in an ongoing, longitudinal, quasi-experimental study protocol, which has been accepted and published in *BMJ Open*.^32^ The upcoming study will outline the structured capture and analysis of the following clinical outcomes, namely:

(1) Anaemia prevalence and aetiology (including Hb, ferritin, transferrin saturation, vitamin B12 and folate levels);
(2) Hb variation (from pre-operative assessment to surgery and discharge);
(3) Iron, vitamin and erythropoiesis-stimulating agent use;
(4) Adherence to PBM protocol interventions, including timing and modality of prehabilitation;
(5) Viscoelastic test-guided transfusion practices (FFP, platelets, 4-factor PCC, fibrinogen);
(6) Use of tranexamic acid and cell salvage;
(7) Red blood cell transfusion rates and units administered;
(8) Length of stay (ICU, ward and overall);
(9) Postoperative complications (cardiac, renal, infectious, etc.);
(10) Mortality, 12-month survival and readmission rates;
(11) Cost-effectiveness metrics.

These data are currently being gathered through an integration between the hospital information system and a digital PBM platform, ensuring the real-time, structured collection of both CROMs and PROMs in alignment with VBHC principles.

In future versions of this work or subsequent manuscripts, we intend to include these outcome metrics and subgroup analyses, which will enable a deeper understanding of the clinical effectiveness of the digital PBM journey and its impact on patient outcomes.

### Personalisation and scalability

This study highlights the ability of digital platforms to personalise care through real-time data integration, bridging gaps in traditional, static, paper-based systems.^13^^,^^31^

The platform's adaptability across specialties along with multilingual capabilities (up to 12 languages), highlights its scalability and broad applicability.

### Broader implications for value-based healthcare

Integrating PROMs within patient pathways supports VBHC principles by improving patient engagement and aligning care delivery with efficiency and effectiveness goals. These findings complement studies on value-based anaesthesiology, where bundled payment systems and accountable care organisations incentivise high-quality care through shared savings.

### Limitations

The single-centre design and short observation period of this study limit its generalisability. Although PROM adherence proportions were initially robust, a decline over time underscores the need for strategies to maintain long-term patient engagement, as sustaining adherence to digital tools remain challenging.^24^ Statistically significant PROM score changes across timepoints were not observed, likely due to the limited sample size included for feasibility purposes.

In addition, reasons not to participate and satisfaction with the participation were not collected in a formal or structured fashion. Moreover, this study does not feature CROMs data in the results to help understand the clinical impact of the adoption of the PROMs digital pathway. A future study will present these insights.^32^

### Future directions

(1) Leveraging artificial intelligence driven analytics to refine personalised care and further integrate anaesthesiology in data-driven VBHC.
(2) Conducting cost-benefit analyses to assess the financial sustainability of digital PROMs collection practices.
(3) Expanding the observation period and incorporating larger, multicentre cohorts.
(4) Extending automated digital PROMs collection to other surgical specialties.

## Conclusion

This study underscores the transformative potential of integrating digital tools within anaesthesiology-driven peri-operative pathways. By enabling real-time monitoring and personalised interventions, the automated digital PROMs collection journey advances the principles of VBHC, setting a benchmark for future innovations in peri-operative care.

## Supplementary Material

Supplemental Digital Content
