# Supplementary material for: Implementation of a patient-reported outcome measure based digital journey and automation tool: A feasibility study in a surgical setting
Source: Eur J Anaesthesiol Intensive Care. 2026 Jun 22;5(4):e0122. doi: 10.1097/EA9.0000000000000122 (PMC13568202; doi:10.1097/EA9.0000000000000122)
Supplement: Supplementary file 1 [file ejaic-5-e0122-s001.pdf]

UNIDADE LOCAL DE SAÚDE
GAIA E ESPINHO

REPÚBLICA PORTUGUESA
SAÚDE

SNS SERVIÇO NACIONAL DE SAÚDE

Patient Blood Management

ULSGE a cuidar da saúde do seu sangue.

CUIDAMOS DE SI

A saúde do seu sangue é importante

A Gestão do sangue do doente – Patient Blood Management (PBM) – consiste numa abordagem **centrada no doente**, **baseada na evidência** que tem como objetivo melhorar o desempenho do doente através da **preservação do seu sangue**.

Envolve a utilização de estratégias médicas e cirúrgicas seguras e eficazes para prevenir, diagnosticar e tratar a anemia; diminuir as perdas hemáticas e hemorragia. **Promove o empoderamento do doente e a qualidade e segurança do seu tratamento**.

Objectivos

- Melhorar a saúde global da nossa população.
- Melhorar o bem-estar dos doentes e a sua qualidade de vida.
- Melhorar o desempenho peri-operatório do doente.
- Diminuir o tempo de internamento hospitalar.
- Minimizar o risco de infeções.
- Ao reduzir a necessidade de transfusões sanguíneas, reduz os efeitos adversos e complicações decorrentes das mesmas.

Sabe qual o seu valor de hemoglobina?

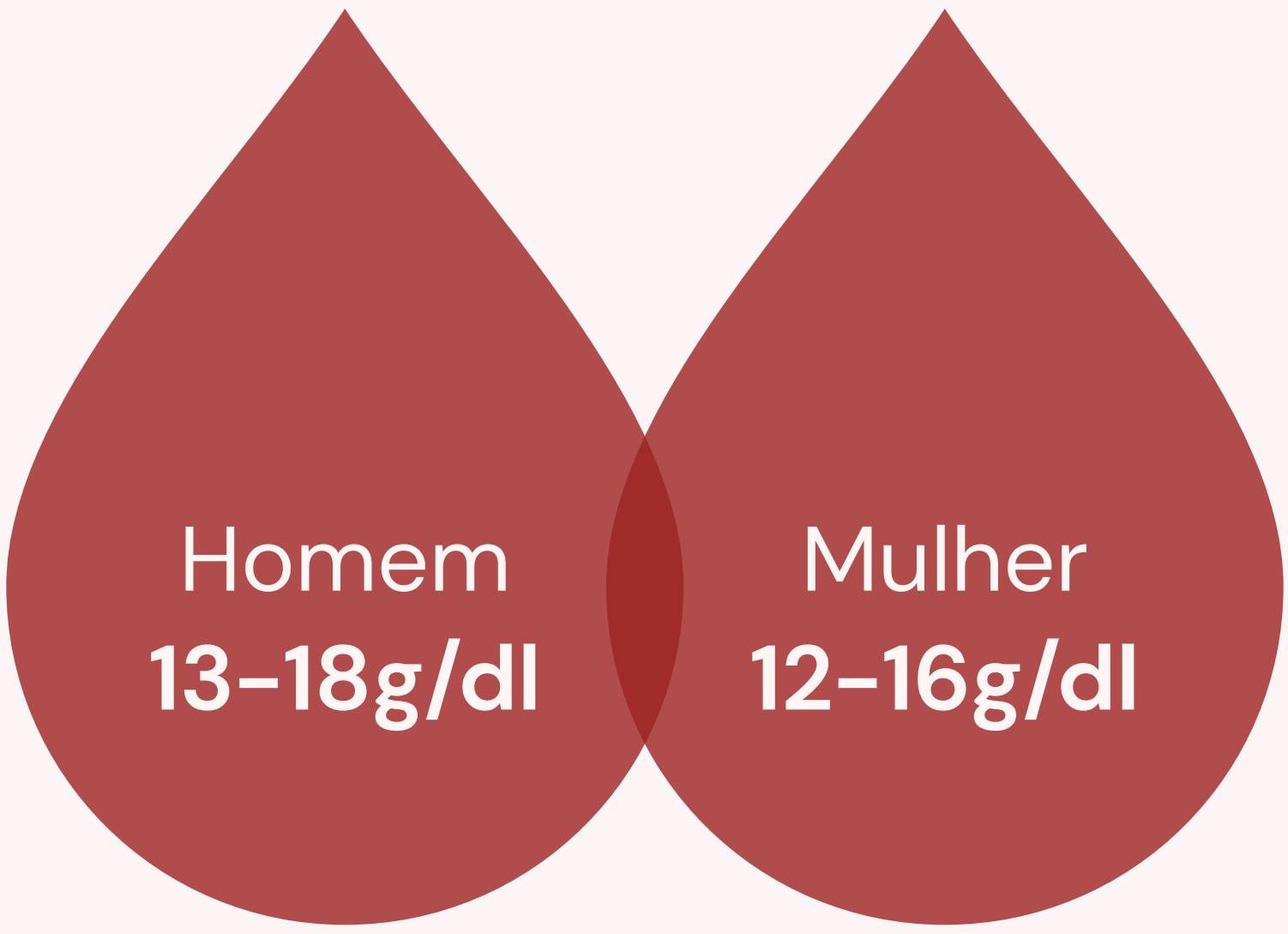

Valores normais de hemoglobina

Quando o nível de hemoglobina é mais baixo que o normal, dizemos que o doente tem anemia.

Como me preparo para a cirurgia?

Assim que for proposto para cirurgia terá de fazer **análises ao sangue**, para saber se tem anemia e/ou défice de ferro, ácido fólico e/ou Vitamina B12. Serão também despistadas alterações da coagulação que podem acarretar maior tendência hemorrágica ou trombótica.

Caso isto se verifique, **poderá ser encaminhado para uma consulta de Hematologia/Imuno-hemoterapia**, onde será avaliada a necessidade de fazer medicação:

Eritropoietina
Hormona que estimula a produção de glóbulos vermelhos

Ferro (oral ou injetável)
Mineral essencial para a formação de glóbulos vermelhos e transporte de oxigénio

Vitamina B12 / ácido fólico
Vitaminas necessárias à produção de glóbulos vermelhos

Estudo de alterações da coagulação permitindo planear a cirurgia de forma mais segura

Com a finalidade de descobrir a causa da anemia poderá ainda justificar-se a **realização de exames adicionais**, quer a nível hospitalar, quer pelo seu médico de família.

Se estiver a tomar **medicamentos que afetem a coagulação do sangue**, o seu médico poderá indicar-lhe que estes **deverão ser suspensos antes da cirurgia**, uma vez que aumentam o risco de hemorragia, por exemplo: ácido acetilsalicílico, clopidogrel, ticagrelor, prasugrel -varfarina, acenocumarol -dabigatrano, apixabano, edoxabano, rivaroxabano.

Deve referir ao seu médico produtos naturais que esteja a tomar.

Estratégias para reduzir a hemorragia durante a cirurgia

Durante a cirurgia, são várias as estratégias adotadas para reduzir as perdas sanguíneas:

Cirurgia minimamente invasiva / técnica cirúrgica meticulosa:
Utilização de técnicas e instrumentos que minimizam as perdas sanguíneas.

Colheita e reinfusão do sangue perdido:
O sangue que é perdido durante a cirurgia é colhido e novamente administrado. Inclui a técnica de cell-saver, que consiste na aspiração do sangue do campo cirúrgico e que é submetido a um processamento do qual se obtém glóbulos vermelhos.

Antifibrinolíticos:
Medicamentos injetáveis que ajudam na coagulação do sangue.

Utilização de testes viscoelásticos:
Permitem gerir de forma precisa as alterações da coagulação.

A escolha das estratégias mais adequadas a cada indivíduo, bem como a avaliação da existência de contra-indicação para cada uma delas, é feita pela equipa médica envolvida na sua cirurgia (cirurgião e anestesiologista).

Qual o meu papel neste processo?

Existem muitas estratégias para otimizar a anemia, alterações da coagulação ou hemorragia que melhoram o seu desempenho no peri-operatório.

Para alguns doentes, a transfusão de sangue pode nunca ser uma opção por motivos médicos, religiosos ou pessoais.

Cada tratamento apenas é administrado após a obtenção do seu consentimento, pelo que é fundamental que cada indivíduo tome uma decisão esclarecida, com o auxílio dos profissionais de saúde.

UNIDADE LOCAL DE SAÚDE
GAIA E ESPINHO

Rua Conceição Fernandes,
4434-502 Vila Nova de Gaia

+351 227 865 100

geral@chvng.min-saude.pt

CUIDAMOS DE SI
