# Supplementary material for: Implementation of a patient-reported outcome measure based digital journey and automation tool: A feasibility study in a surgical setting
Source: Eur J Anaesthesiol Intensive Care. 2026 Jun 22;5(4):e0122. doi: 10.1097/EA9.0000000000000122 (PMC13568202; doi:10.1097/EA9.0000000000000122)
Supplement: Supplementary file 2 [file ejaic-5-e0122-s002.pdf]

Example of the dynamic dashboard individual view (patient-level PROM evolution).

This example patient needed pre-operative optimisation. A clinical aggravation was detected, which motivated a change in surgical priority. A minithoracotomy was performed.

Patient-level Clinical View

PROMs CROMs

Quality of Life (SF-36 Questionnaire)

Scores Evolution by Component (0-100): Higher score corresponds to higher quality of life

Physical Component

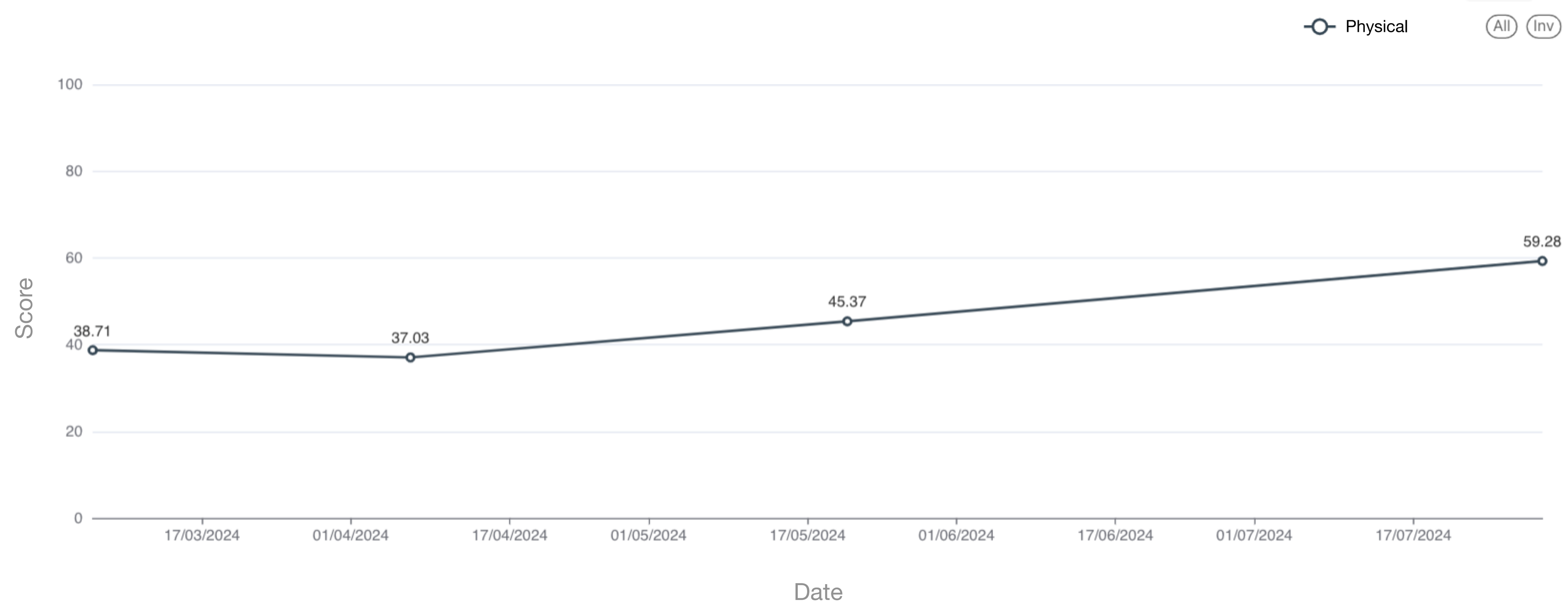

Mental Component

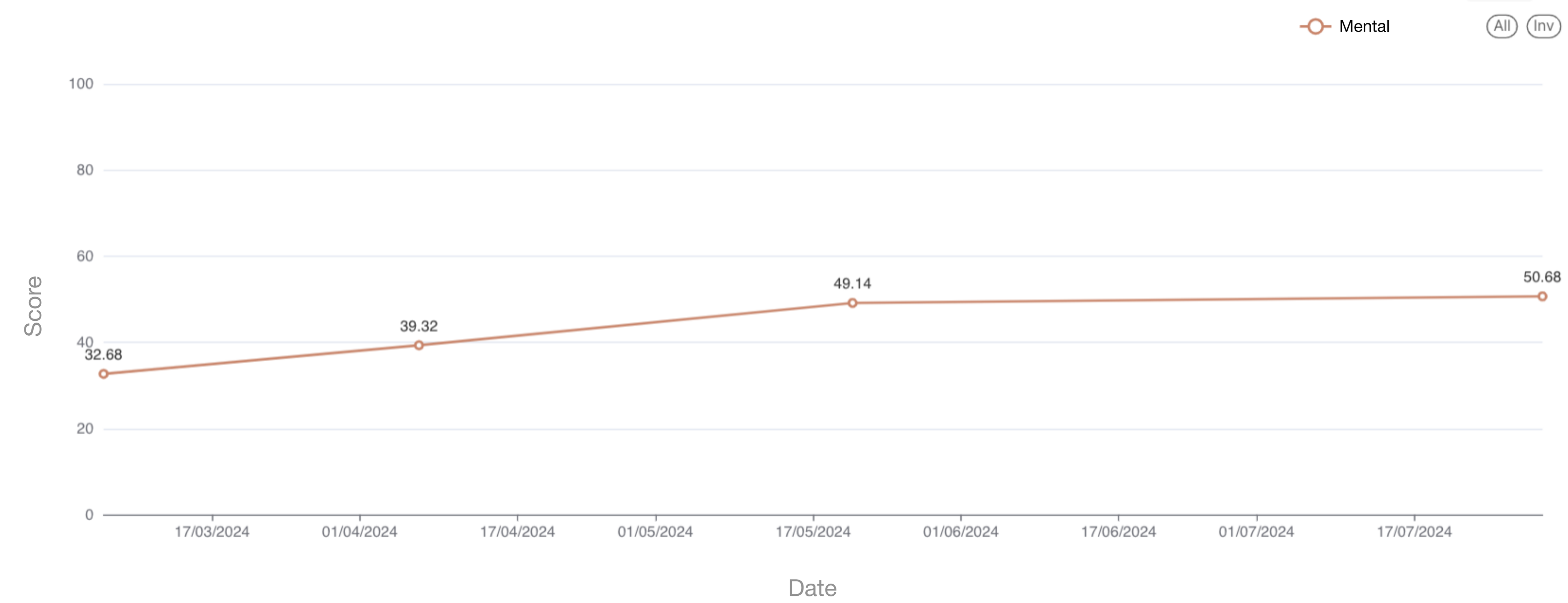

Pain Evaluation (BPI Questionnaire)

0-10: Higher score corresponds to higher Pain Severity/Interference

Pain Severity Index

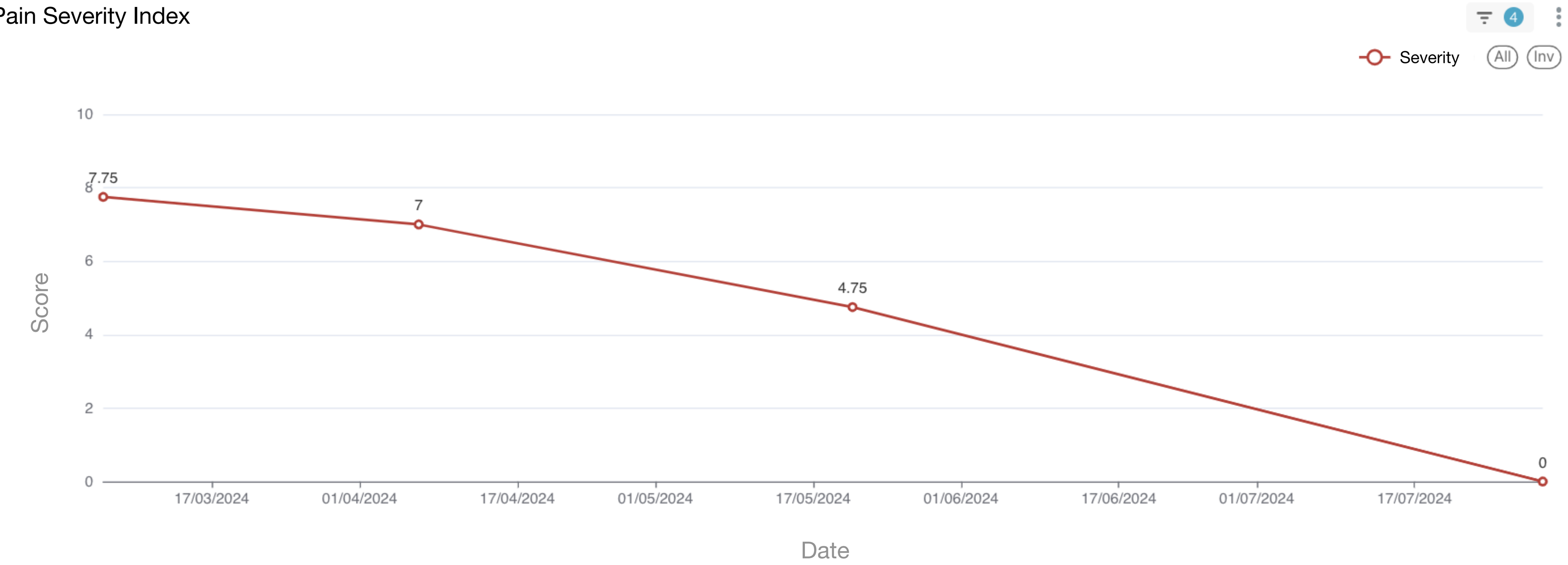

Pain Interference Index

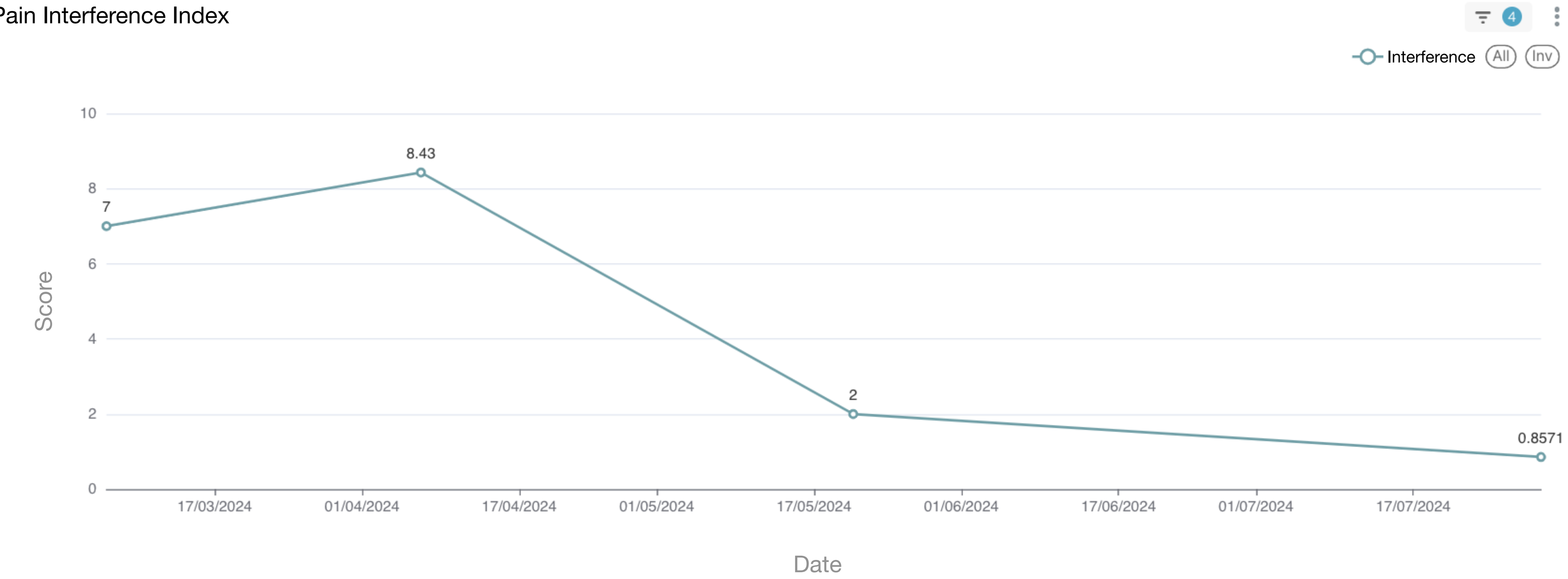

Depression (PHQ-9 Questionnaire)

0-4: Minimal Depression, 5-9: Mild Depression; 10-14: Moderate Depression, 15-19: Moderately Severe Depression, 20-27: Severe Depression

Score and Interpretation

Process Number	Patient ID	Interpretation	Score	Completion Date
Confidential Information		Minimal depression	2	2024-07-30
		Minimal depression	3	2024-05-21
		Mild depression	8	2024-03-06

Use of Health Services External to the Hospital

Process Number	Patient ID	Use of Health Services External to the Hospital	Completion Date
Confidential Information		No	2024-07-30
		No	2024-05-21
