# Supplementary material for: Implementation of a patient-reported outcome measure based digital journey and automation tool: A feasibility study in a surgical setting
Source: Eur J Anaesthesiol Intensive Care. 2026 Jun 22;5(4):e0122. doi: 10.1097/EA9.0000000000000122 (PMC13568202; doi:10.1097/EA9.0000000000000122)

Patient’s View

The patient receives notifications by e-mail and/or SMS.

Notifications contain information, in patient’s native language, that might be relevant for the hospital clinical team to share (for example, what is Patient Blood Management and what to expect).

Example of a Welcome message with educational content:

1. The patient receives an e-mail (and/or SMS) and reads the information.

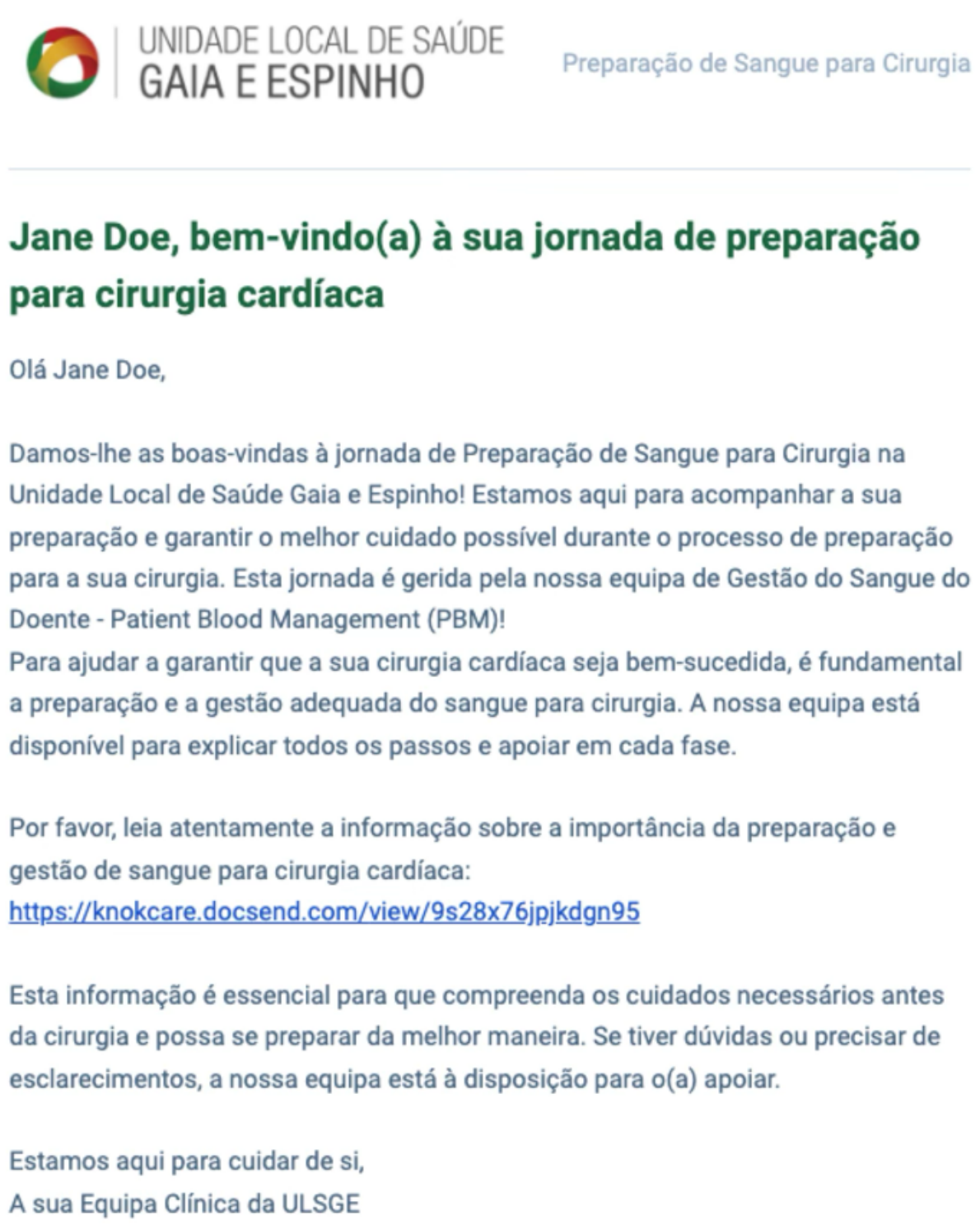

UNIDADE LOCAL DE SAÚDE GAIA E ESPINHO

Preparação de Sangue para Cirurgia

Jane Doe, bem-vindo(a) à sua jornada de preparação para cirurgia cardíaca

Olá Jane Doe,

Damos-lhe as boas-vindas à jornada de Preparação de Sangue para Cirurgia na Unidade Local de Saúde Gaia e Espinho! Estamos aqui para acompanhar a sua preparação e garantir o melhor cuidado possível durante o processo de preparação para a sua cirurgia. Esta jornada é gerida pela nossa equipa de Gestão do Sangue do Doente - Patient Blood Management (PBM)!
Para ajudar a garantir que a sua cirurgia cardíaca seja bem-sucedida, é fundamental a preparação e a gestão adequada do sangue para cirurgia. A nossa equipa está disponível para explicar todos os passos e apoiar em cada fase.

Por favor, leia atentamente a informação sobre a importância da preparação e gestão de sangue para cirurgia cardíaca:
https://knokcare.docsend.com/view/9s28x76jpjkdgn95

Esta informação é essencial para que compreenda os cuidados necessários antes da cirurgia e possa se preparar da melhor maneira. Se tiver dúvidas ou precisar de esclarecimentos, a nossa equipa está à disposição para o(a) apoiar.

Estamos aqui para cuidar de si,
A sua Equipa Clínica da ULSGE

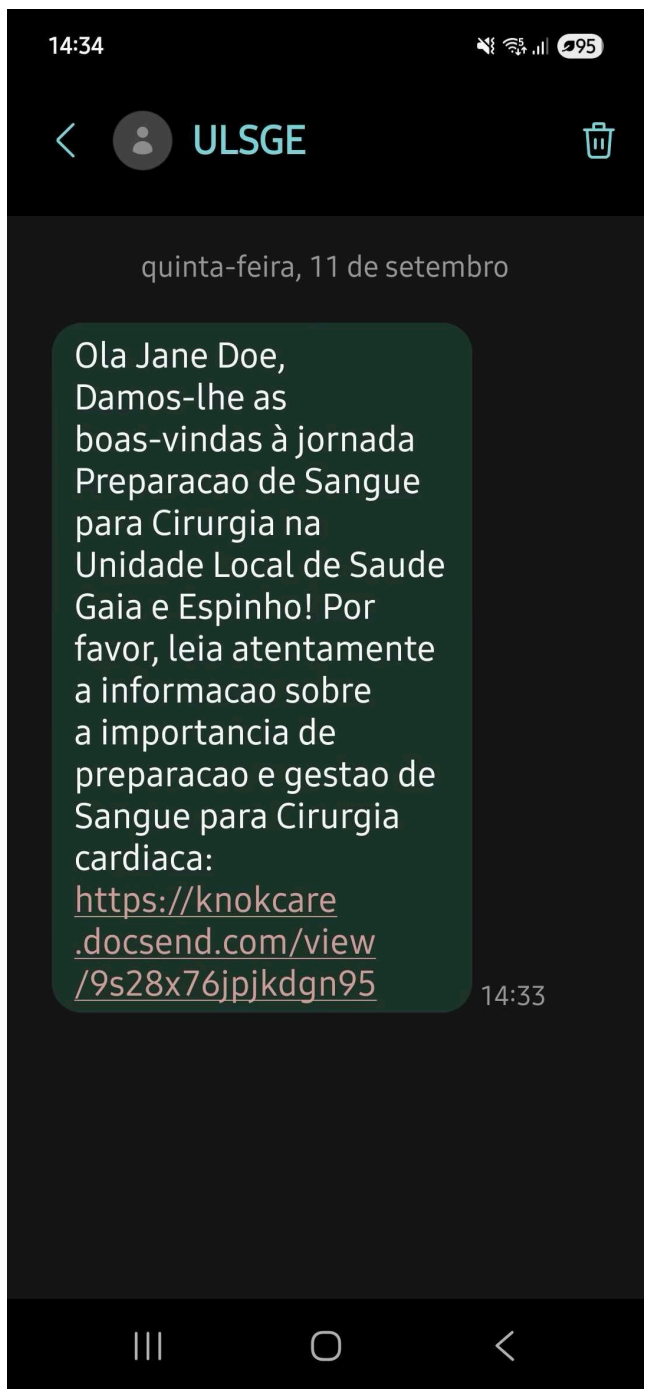

14:34

ULSGE

quinta-feira, 11 de setembro

Ola Jane Doe,
Damos-lhe as boas-vindas à jornada Preparacao de Sangue para Cirurgia na Unidade Local de Saude Gaia e Espinho! Por favor, leia atentamente a informacao sobre a importancia de preparacao e gestao de Sangue para Cirurgia cardiaca:
https://knokcare.docsend.com/view/9s28x76jpjkdgn95

14:33

2. By clicking on the hyperlink that is present in the e-mail body or SMS, the content automatically opens in the computer or mobile phone, respectively.

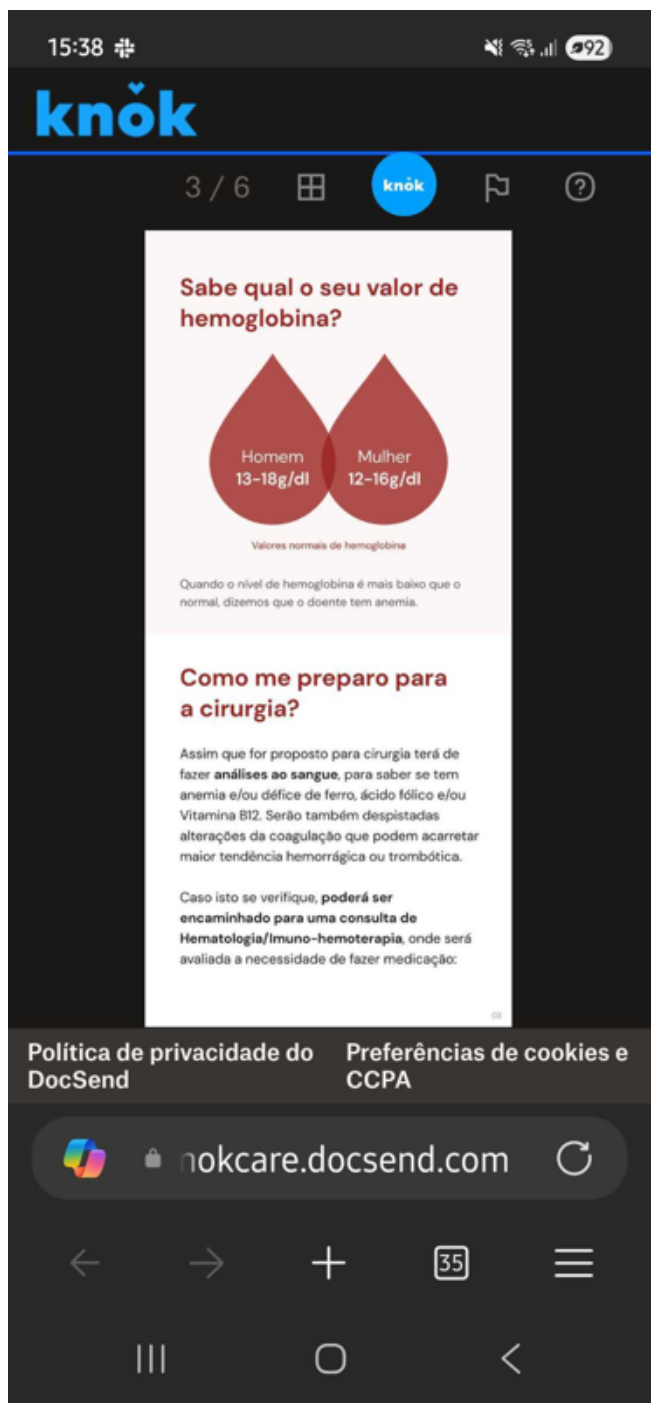

Patients also receive notifications to complete digital questionnaires.

Example of a Message to complete a digital questionnaire:

1. <u>The patient receives an e-mail (and/or SMS) and reads the information.</u>

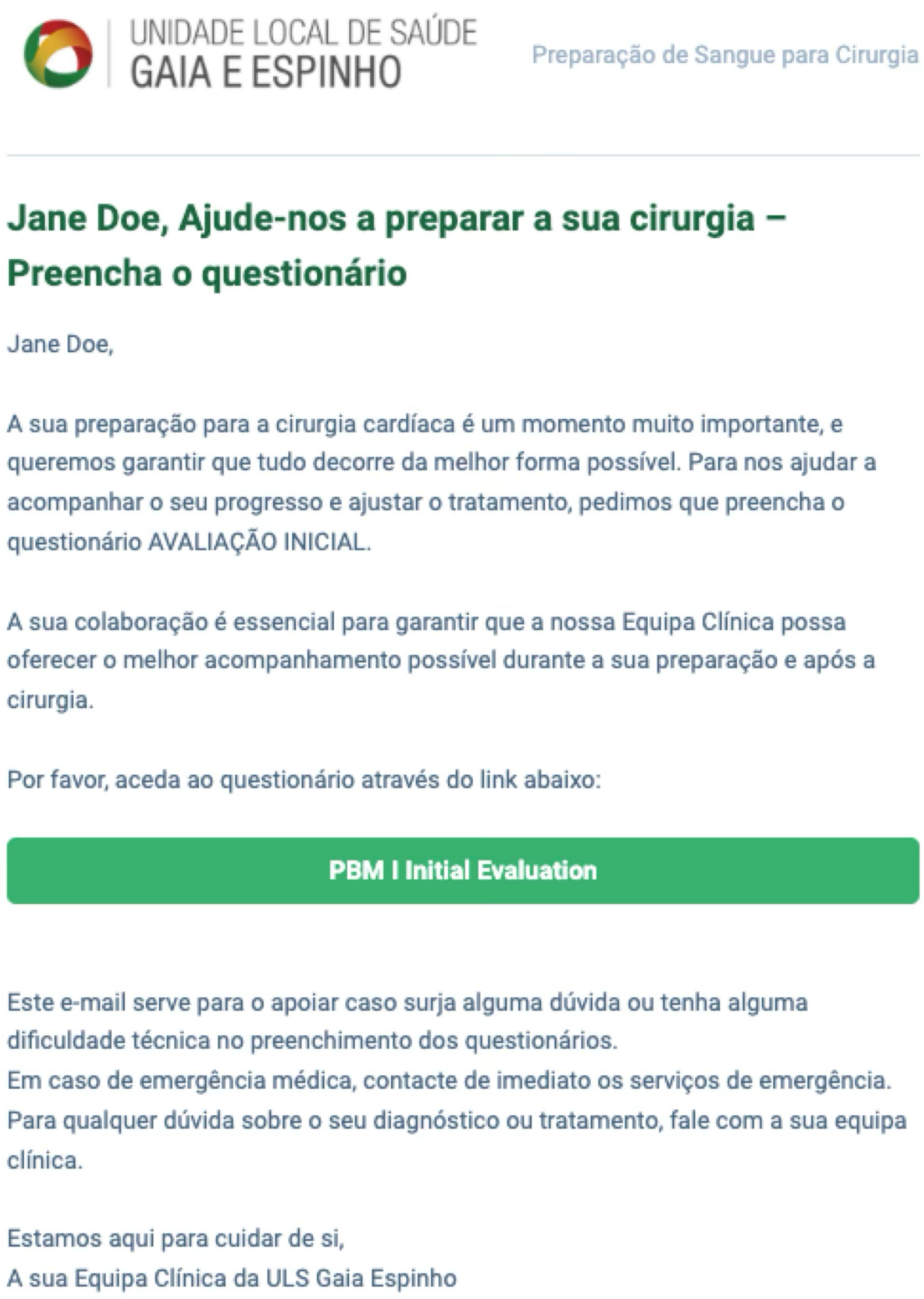

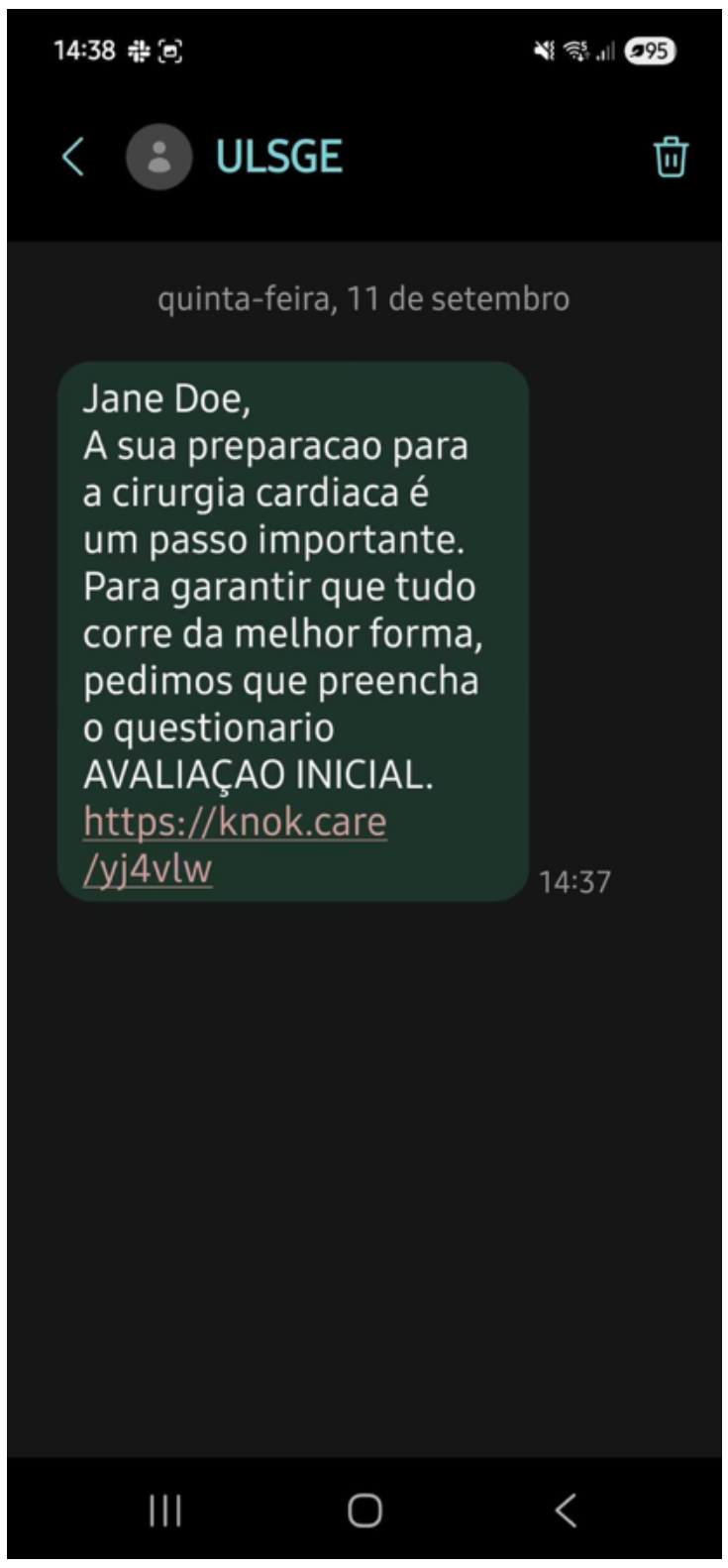

2. <u>By clicking on the green rectangle that is present in the e-mail body, or the hyperlink that is present in the SMS, the digital questionnaire automatically opens for completion in the computer or mobile phone, respectively.</u>

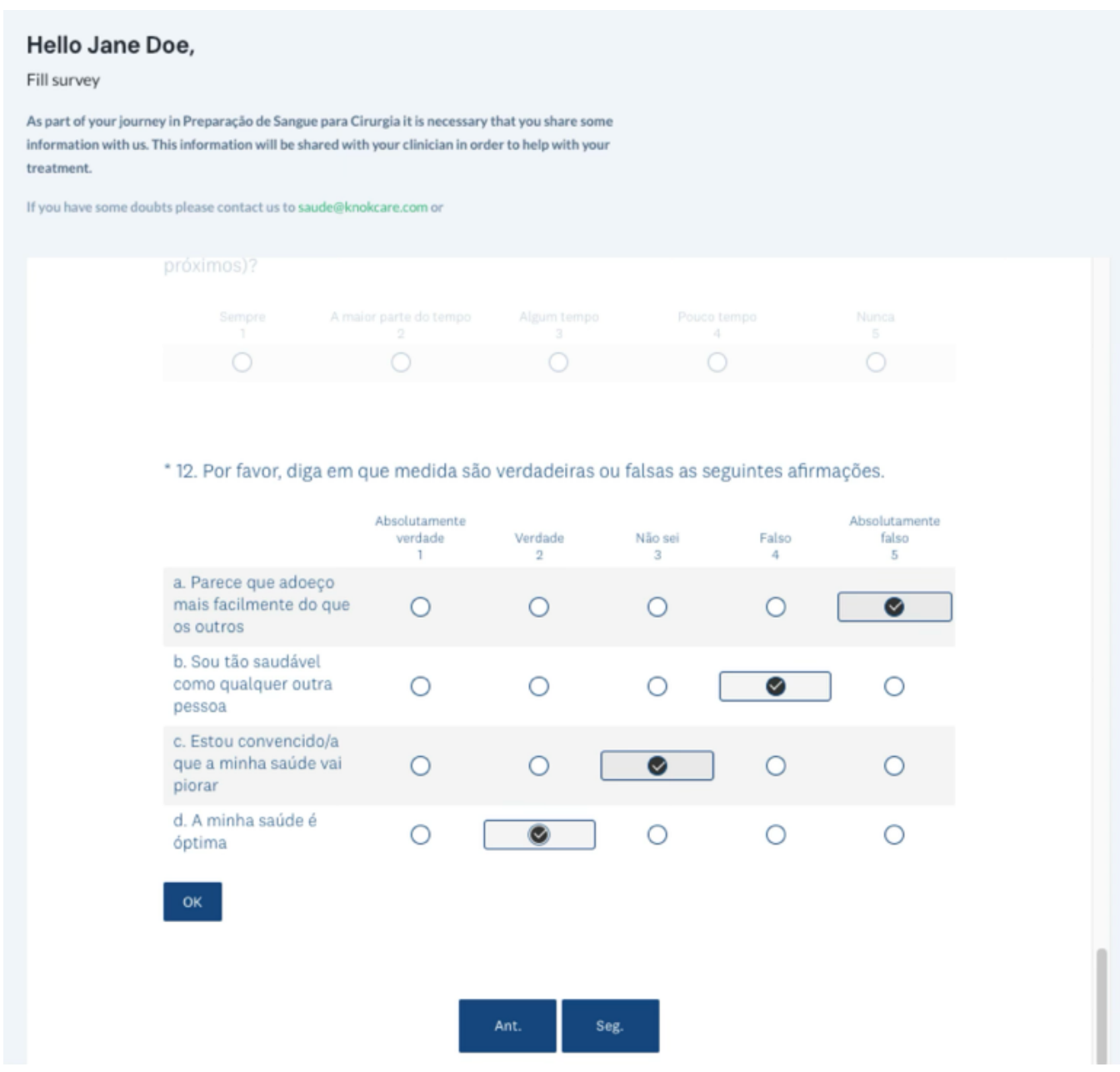

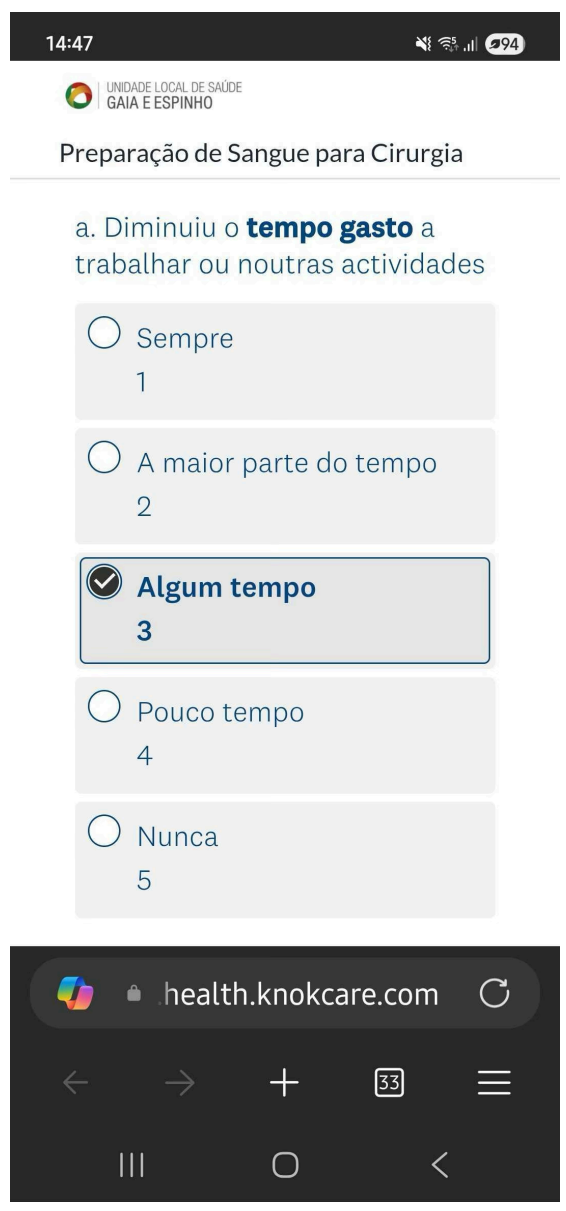

Supplement: Supplementary file 3 [file ejaic-5-e0122-s003.pdf]
